# Trial of Zolpidem, Eszopiclone, and Other GABA Agonists in a Patient with Progressive Supranuclear Palsy

**DOI:** 10.1155/2014/107064

**Published:** 2014-09-16

**Authors:** Andrew Young Chang, Erica Weirich

**Affiliations:** ^1^Department of Internal Medicine, Stanford University Medical Center, 300 Pasteur Drive, Palo Alto, CA 94305, USA; ^2^Palo Alto Medical Foundation, 795 El Camino Real, Palo Alto, CA 94301, USA

## Abstract

Progressive supranuclear palsy (PSP) is a progressive, debilitating neurodegenerative disease of the Parkinson-plus family of syndromes. Unfortunately, there are no pharmacologic treatments for this condition, as most sufferers of the classic variant respond poorly to Parkinson medications such as levodopa. Zolpidem, a gamma aminobutyric acid (GABA) agonist specific to the *α*-1 receptor subtype, has been reported to show improvements in symptoms of PSP patients, including motor dysfunction, dysarthria, and ocular disturbances. We observed a 73-year-old woman with a six-year history of PSP, who, upon administration of a single 12.5 mg dose of sustained-release zolpidem, exhibited marked enhancements in speech, facial expressions, and fine motor skills for five hours. These results were reproduced upon subsequent clinic visits. In an effort to find a sustainable medication that maximized these beneficial effects while minimizing side effects and addressing some of her comorbid neuropsychological conditions, a trial of five other GABA receptor agonists was performed with the patient's consent, while she and her caregivers were blinded to the specific medications. She and her caretakers subsequently reported improvements, especially visual, while on eszopiclone, and, to a lesser degree, temazepam and flurazepam.

## 1. Introduction

Progressive supranuclear palsy (PSP) is a progressive, incapacitating neurodegenerative disease of uncertain etiology. It is diagnosed clinically, with key features of axial rigidity, downgaze palsy, and frequent falls due to gait ataxia [1]. PSP is a member of the Parkinson-plus family of syndromes and, on pathology, exhibits gliosis, neuronal loss, and neurofibrillary tangles primarily in the basal ganglia, subthalamic nucleus, several brainstem nuclei, and dentate nucleus of the cerebellum [2].

Although the histopathological changes are most striking in subcortical structures, studies have suggested that the cerebral cortex is significantly affected, especially in cortical interneurons containing gamma aminobutyric acid (GABA) benzodiazepine/GABA_A_ receptors [2]. In addition, a decreased number of neurons have been found in the caudate nucleus, ventral striatum, and external and internal palladium of several PSP patients [3]. The progressive loss of these GABAergic structures is believed to result in the motor symptoms of the disorder.

Currently, there are no pharmacologic treatments for PSP, as early trials with Parkinson medications such as levodopa failed to show clinical improvement among patients. Experimental attempts with glutamate receptor blockers, adrenergic antagonists, and acetylcholinesterase inhibitors have similarly been unable to relieve the neuromuscular impairments of PSP [4–6]. Given the importance of GABAergic neurons in the pathogenesis of the disease, however, drugs acting at GABA receptors may help alleviate some of the deficits of PSP. Zolpidem, a nonbenzodiazepine hypnotic, is specific for the *α*-1 subtype of the GABA_A_ receptor. In particular, these receptors are found in highest density in the internal palladium and may be associated with activity of the thalamus and motor cortex [7].

The potential of this medication has led to several small case studies investigating its possible efficacy: a double-blind placebo controlled crossover study with ten PSP patients found improved motor and ocular function in patients on a single dose of zolpidem compared to levodopa or placebo [8]. A case study following this publication noted extinguishment of the therapeutic effect over time of a patient taking zolpidem IR (immediate release) for four weeks, while another described sustained improvement of motor and bulbar symptoms over six months using zolpidem CR (sustained release) [9, 10]. Zolpidem has also been reported to have unexpected therapeutic benefits for cognitive and motor deficits in a variety of other neurologic conditions such as stroke and anoxic brain injury [11–15].

As of yet, however, there exist no reports of other GABA agonist medications showing improvement in PSP patients. Given that the GABA receptor has multiple subtypes with various functions, trials of these may help in both identifying effective new medications while proposing additional pathways in the pathogenesis of the disease.

## 2. Case Presentation

We observed a 73-year-old woman with a six-year history of PSP, who upon challenge with a single 12.5 mg dose of zolpidem CR exhibited marked improvements in speech, facial expressions, and fine motor skills via both subjective observation and a standardized PSP Rating Scale (Foundation for PSP, CBD and Related Brain Disease: www.psp.org).

The patient had a long history of worsening slurred speech, poor balance, frequent falls, and difficulty writing over a six-year period. Her symptoms went undiagnosed for many years, however, as she did not fit the criteria for typical Parkinson disease and was unsuccessfully trialed with carbidopa-levodopa. Her symptoms progressed over this period to the point that she became wheelchair-bound and dependent on a home health aide to complete activities of daily living (ADLs). Eventually, she was diagnosed with PSP based on meeting the official clinical criteria proposed by the National Institute of Neurological Disorders and Stroke (NINDS) and the Society for PSP (SPSP). During this time, the patient developed depression, anxiety, and insomnia and was initially prescribed zolpidem IR as a sleep aid. The inefficacy of this preparation over time, however, led to a switch to zolpidem CR. After two months of taking the new medication, the patient and her husband were surprised that her ability to speak and move was strikingly improved with administration of zolpidem. She also displayed significant psychomotor activation, increased motivation to perform tasks, and lack of nighttime somnolence. Thereafter, the patient's family noted that these effects occurred nightly for a month.

The authors, upon a subsequent clinic encounter, verified these results when she took 12.5 mg of zolpidem CR two hours prior to the visit. Her functional enhancement lasted for approximately five hours. Due to the impressive quality of life improvement reported by the patient, along with the increasingly severe decline in functional status resulting from the disease, she desired to find a sustainable medication that maximized the beneficial effects of zolpidem while minimizing side effects and addressing some of her comorbid neuropsychological conditions. As such, the patient agreed to a trial of five other GABA receptor agonists (Table 1). The single dose of each agonist was selected as the maximum safe long-term use dosage for the subject. She also underwent a weekend “washout” off all GABA agonists prior to the trial.

Each test day, the patient was given the day's medication by her caretaker while blinded (due to her functional impairments, the subject had her caretaker place each pill in her mouth without revealing its identity (this individual was also blinded to the day's trial PSP medication)). The patient and her husband (who was also blinded to the day's drug) then recorded their subjective impressions of the patient's activity, including speech, eating, salivation, vision, writing, drowsiness on the medication, and emotional state. Her husband was also trained to administer Folstein's minimental status exam [16], which he did every day as an objective measure of her cognitive function. Each week, the patient returned to clinic for a physical examination and evaluation via the PSP Rating Scale (Foundation for PSP, CBD and Related Brain Disease: www.psp.org).

### 2.1. Outcome/Results

At baseline (without any medications), the patient was observed to exhibit the significant and debilitating symptoms consistent with PSP. These included a stooped, left-leaning posture, cogwheel rigidity on passive range of motion, and a resting tremor of the right hand. Her face had a wide-eyed “stare,” with a slightly protruding tongue and considerable sialorrhea. She was unable to walk and had severe bradykinesia of all extremities. The patient also had a slowed downward gaze, with tracking faster left-to-right than right-to-left. She often went about her days with one eye closed due to disruptive double vision. Her dysarthria prevented her from intelligible vocal speech, leaving her only the ability to make monosyllabic sounds with poor enunciation. She communicated primarily with gestures and a letter/word board. She also had dysphagia, resulting in frequent coughing with even the consumption of clear liquids.

Two hours following a 12.5 mg dose of zolpidem CR, the patient displayed a remarkable improvement in many of these symptoms at a clinic visit. Her posture straightened and her stare was less pronounced. Her tongue was no longer protruding, and there was a significant decrease in drooling. Though she was still too weak to ambulate on her own, her spontaneous movements were increased, as she fidgeted with her fingers, rocked herself with her legs, and pointed and gestured freely. Repetitive thumb-finger pinching movements were improved from baseline. Interestingly, the patient had an exaggerated 4+ hyperreflexia of the biceps, brachioradialis, and patellar reflexes bilaterally while on the medication, which was not noticeable at baseline.

**Figure 1:**
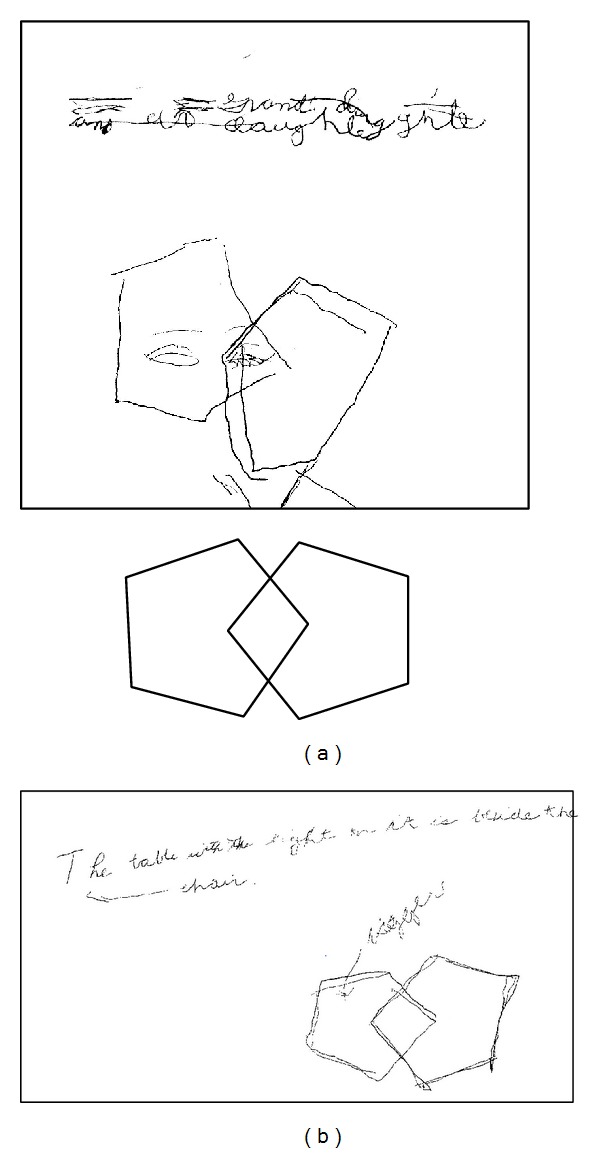
Writing/drawing samples from patient. A writing sample and attempt at copying the Folstein MMSE intersecting pentagon figure. The left panel shows an attempt at both in the absence of medication. The right panel shows subsequent improvement when taking zolpidem CR. The original figure from the Folstein document is reproduced above.

The patient was able to speak in intelligible complete sentences with a full vocabulary and normal content, though her speech was still slightly slurred. Facial expressiveness was greatly expanded (e.g., visible changes during thinking, laughing, frustration, smiling, and whispering). Fine motor control was improved, as demonstrated in her quality of writing and figure drawing (Figure 1). She also noted greater motivation to converse, write, and move herself. She denied feelings of somnolence or confusion, instead expressing that she felt more alert and active.

After this encounter, the patient was placed on an every-other-day trial of different GABA agonists, including zolpidem CR in two forms (one pill 12.5 mg, two pills 6.25 mg each), eszopiclone, alprazolam, temazepam, flurazepam, and triazolam. In addition, she was given a one-time dose of tizanidine, an *α*-2 adrenergic agonist, as a muscle relaxant for the painful muscle spasms she was developing secondary to her disease. The self-reported changes experienced by the patient and her husband, along with Folstein MMSE scores, are summarized in Table 2.

The patient's greatest response to treatment was with zolpidem CR, on which she showed an improvement in PSP Rating Score from 62 at baseline to 58. In addition, her best MMSE score of 28/28 was recorded while on zolpidem CR, compared to her lowest MMSE scores of 23/28 and 25/28 without medication. Interestingly, the patient also noted alleviation of certain PSP deficits on her eszopiclone trial. Though her speech improvement was shorter-lived than on her zolpidem test days, she reported greater relief of her double vision. She also noticed enhancement of her speech, though only for a short duration, on temazepam and flurazepam. Alprazolam and tizanidine had no effects on her PSP symptoms.

## 3. Discussion

Although three case studies noting the improvement of PSP patients on zolpidem of various preparations have been noted in the literature, to our knowledge, this is the first time other GABAergic agents have been reported as alleviating the symptoms of the disorder. It is worth noting that all tested GABA agonist medications are active at the *α*-1 subtype of the GABA_A_ receptor, though to various degrees of specificity [17]. In particular, zolpidem and eszopiclone are more specific for the *α*-1 subunit than their benzodiazepine counterparts. It is this specificity which is believed to result in the lesser side-effect profile of these “z-drugs,” with better tolerability, reduced muscle relaxant properties, and lack of withdrawal effects. We were able to see the greatest improvements in symptoms (for the longest duration) on these two drugs. Interestingly, though both are *α*-1 subunit specific, the patient noted that her speech was better when taking zolpidem, while her vision was better on eszopiclone. Small differences in receptor site specificity may account for these distinctions [18].

Temazepam, flurazepam, and triazolam are considered “traditional hypnotic benzodiazepines” with similar functions. Though they, like zolpidem and eszopiclone, bind to GABA_A_ receptors, they lack *α* subunit specificity, binding to *α*-1, 2, 3, and 5 alike. Perhaps it is this difference that accounted for the fact that our patient reported modest improvements on these drugs, though not to the extent noted on zolpidem. Alprazolam, a benzodiazepine with effects on non-GABA_A_ receptors, had no noticeable effects on the subject [19]. As expected, the patient noted no improvements with the non-GABAergic agent tizanidine.

Clinically, the results seen by our patient most resembled those reported by Cotter and colleagues. In both studies, the use of the CR preparation of zolpidem was associated with sustained therapeutic effects for over five months in PSP patients. A prior case report by Mayr and colleagues using the IR preparation of zolpidem noted an extinguishment of efficacy after four weeks of treatment. Interestingly, our patient then had a period of time several months after the initial investigation during which she did not take zolpidem. As expected, her neurological deficits worsened back to baseline. Subsequent resumption of the medication was associated with a return in lost function as described above, suggesting the effect of the drug was durable and reproducible. Furthermore, both the Cotter report and ours found that there was an initial latency period of two months during which the patients took zolpidem CR but did not experience any symptomatic improvements. We agree with their hypothesis that this delay prior to any observed effects may reflect the need for the drug to overcome a threshold level of GABAergic activity in the affected brain regions before clinical improvements can be seen.

A review of the literature revealed that PSP is not the only condition that has benefited from trials of zolpidem. Both transient and sustained improvements have been reported in stroke-associated aphasia, postanoxic spasticity, spinocerebellar ataxia, and semicoma from anoxic brain injury [11–15]. One study also supported the assertion that the medium of action is *α*-1 subunit specific: though zolpidem could arouse a patient from a semicoma state, diazepam, a non-*α*-specific benzodiazepine, had no effect [14]. It is worth noting that, in all these conditions, zolpidem resulted in a restoration of function or increased activation/arousal, contrary to its indicated use as a hypnotic/sedative. Several studies have examined these patients with single-photon emission computed tomography (SPECT) while on the drug and revealed significantly increased cerebral blood flow to the areas of brain injury responsible for disease symptoms [11, 13, 14]. It is possible that a similar reversal of dynamic diaschisis near degenerated regions of the PSP brain could account for zolpidem's positive effects.

Based on our observations and investigation, we deemed the functional quality-of-life benefits provided by zolpidem significant enough to warrant regular daytime dosing for our study patient. She was prescribed 12.5 mg of zolpidem CR each morning and has continued to note sustained improvement at five months following the conclusion of the trial. The specific therapeutic mechanism of action of such *α*-1 subunit specific GABA agonists in PSP has yet to be elucidated, and further studies will be necessary to better understand both different GABA_A_ subunits and how they are pharmacologically activated in different combinations. A larger cohort of patients, with more objective imaging data such as PET or SPECT scans, may aid in this endeavor. Hopefully, these initial insights may pave the way for the development of new, more potent GABAergic medications to treat PSP and other debilitating neurological conditions.

## Figures and Tables

**Table 1:** Medications by trial day and dose.

Trial day	Name of medication	Dose
0	Alprazolam	3 mg
1	Zolpidem CR	12.5 mg
3	Eszopiclone	3 mg
6	Zolpidem CR	2 tablets 6.25 mg each
8	Tizanidine	4 mg
10	Temazapam	7.5 mg
12	Zolpidem CR	12.5 mg
14	Flurazepam	30 mg
17	Triazolam	0.25 mg

**Table 2:** Impressions and effects of medication trials.

Trial day	Name of medication	Dose	Effects (comments)	MMSE	PSP
0	Alprazolam	3 mg	No improvements, some sedation.	—	—
1	Zolpidem CR	12.5 mg	Excellent. Good speech, strong desire to converse.	—	58
3	Eszopiclone	3 mg	Speech improved, though not as much as on zolpidem. Double vision markedly improved. Effects extinguished more quickly than zolpidem.	27	—
4	No medications		—	23	—
6	Zolpidem CR	2 × 6.25 mg	Best day. Good speech.	28	—
8	Tizanidine	4 mg	None.	27	61
10	Temazapam	7.5 mg	Speech improved slightly. Effects diminished quickly (1.5 hrs) and patient experienced sedation.	28	—
11	No medications		—	25	—
12	Zolpidem CR	12.5 mg	Speech improved for five hours.	—	—
14	Flurazepam	30 mg	Speech improved slightly, but effects diminished quickly and patient experienced confusion.	27/27 (Scored out of 27)	—
15	No medications	—	—	25	62
17	Triazolam	0.25 mg	Speech improved slightly. Effects diminished quickly and patient experienced some sedation.	27	—

Table describing the patient's response to the various medications. MMSE: Folstein's minimental status exam, scored out of 28 points; higher score denotes higher cognitive function. PSP: progressive supranuclear palsy rating scale (Foundation for PSP, CBD and Related Brain Disease), scored out of 100 points; lower score denotes better symptoms and higher functional status.
